# 3D bioprinting of bicellular liver lobule-mimetic structures via microextrusion of cellulose nanocrystal-incorporated shear-thinning bioink

**DOI:** 10.1038/s41598-020-77146-3

**Published:** 2020-11-26

**Authors:** Yun Wu, Andrew Wenger, Hossein Golzar, Xiaowu (Shirley) Tang

**Affiliations:** Department of Chemistry and Waterloo Institute for Nanotechnology, University of Waterloo, 200 University Ave West, Waterloo, ON N2L 3G1 Canada

**Keywords:** Nanoscale materials, Biomaterials, Biomedical engineering, Nanoscience and technology

## Abstract

3D bioprinting of living cellular constructs with heterogeneity in cell types and extra cellular matrices (ECMs) matching those of biological tissues remains challenging. Here, we demonstrate that, through bioink material design, microextrusion-based (ME) bioprinting techniques have the potential to address this challenge. A new bioink employing alginate (1%), cellulose nanocrystal (CNC) (3%), and gelatin methacryloyl (GelMA) (5%) (namely 135ACG hybrid ink) was formulated for the direct printing of cell-laden and acellular architectures. The 135ACG ink displayed excellent shear-thinning behavior and solid-like properties, leading to high printability without cell damage. After crosslinking, the ACG gel can also provide a stiff ECM ideal for stromal cell growth. By controlling the degree of substitution and polymer concentration, a GelMA (4%) bioink was designed to encapsulate hepatoma cells (hepG2), as GelMA gel possesses the desired low mechanical stiffness matching that of human liver tissue. Four different versions of to-scale liver lobule-mimetic constructs were fabricated via ME bioprinting, with precise positioning of two different cell types (NIH/3T3 and hepG2) embedded in matching ECMs (135ACG and GelMA, respectively). The four versions allowed us to exam effects of mechanical cues and intercellular interactions on cell behaviors. Fibroblasts thrived in stiff 135ACG matrix and aligned at the 135ACG/GelMA boundary due to durotaxis, while hepG2 formed spheroids exclusively in the soft GelMA matrix. Elevated albumin production was observed in the bicellular 3D co-culture of hepG2 and NIH/3T3, both with and without direct intercellular contact, indicating that improved hepatic cell function can be attributed to soluble chemical factors. Overall, our results showed that complex constructs with multiple cell types and varying ECMs can be bioprinted and potentially useful for both fundamental biomedical research and translational tissue engineering.

## Introduction

Three-dimensional (3D) printing is an additive manufacturing process that fabricates 3D architectures layer by layer with various materials, such as plastics, metal, ceramics, and polymers^1–3^. Bioprinting, a subset of 3D printing, holds promise for depositing biomaterials and cells precisely to create heterogeneous tissue-mimetic constructs that promote cell–cell and cell-extracellular matrix (ECM) interactions in a 3D environment which are absent in two-dimensional (2D) cell culture systems^4–6^. Among the different bioprinting techniques such as extrusion-based, light-induced, and inkjet-based methods^7–9^, the microextrusion-based (ME) 3D bioprinting technique has attracted much attention due to its ease of use and the potential to accommodate a wide range of bioink viscosity, offer high cell loading density, and take use of multiple polymerization methods^10–13^.

Although there has been a rapid rise in ME bioprinting research, bioink materials and methods remain limited. Ideal bioinks must meet the requirements for printability, in addition to fulfilling the essential properties required for tissue engineering, such as biodegradability, biocompatibility, cell attachments, and comparable mechanical strength to human tissues^14–16^. Shear-thinning is considered as one of the most important bioink properties for ME since it determines the ultimate printability (e.g. resolution, pattern fidelity) by preventing clogging and reducing shear stress that can lead to cell damages^10,13^. Furthermore, to obtain constructs with a high resolution and a high aspect ratio, the printed scaffold should be mechanically strong enough to avoid collapsing. Instant gelation after deposition or partially pre-gelled hydrogels are also preferred to ensure high structural integrity^17,18^.

In this work, we incorporated alginate, cellulose nanocrystal (CNC), and gelatin methacryloyl (GelMA) to create a hybrid bioink (ACG) and investigated its rheological and mechanical properties. It has been shown that the incorporation of nanoparticles and nanofibers into biopolymers, such as CNC into alginate, can induce favorable shear-thinning behavior^19^. However, our previous study showed that CNC/alginate hydrogel cannot provide a suitable microenvironment for cell proliferation. In particular, it lacks cell attachment sites. Thus, an auxiliary material must be incorporated into the CNC/alginate to increase its bioactivity. Naturally-derived proteins, such as gelatin, collagen, and fibrinogen, are common materials that have been utilized in tissue engineering to promote cell growth^20–22^. Among these materials, gelatin has the advantages of low cost and high purity^23^. However, gelatin tends to liquefy at high temperatures (~ 37 °C), dramatically hindering its application for bioink development. Hence, GelMA derived from gelatin with methacrylamide and methacrylate groups has been used to ensure easy solidification of the printed constructs via UV crosslinking^24^. Moreover, the physical properties of crosslinked GelMA hydrogel can be easily tuned by tailoring the degree of substitution. It has been found that hydrogels made of low concentrations of GelMA with a low degree of modification can improve the cell viability and facilitate cellular organization^25,26^. Here, we designed two types of bioinks, namely the ACG hybrid ink and a low concentration GelMA ink. To evaluate the printability of our bioinks and their efficacy in mimicking the native cellular microenvironment, liver-lobule mimicking structure was chosen as a model. Liver-lobules have well defined geometry that can be printed to scale. In-vitro liver tissue models are in great demand for disease modeling, drug discovery, and clinical applications^27,28^. Recently, multiple literatures have demonstrated the fabrication of liver tissues using various methods and materials, including biofabricating liver lobule-mimetic constructs via dynamic optical projection stereolithography and a piece of liver tissue via ME bioprinting^29–31^. Taken together, our results showed that, through bioink material design, ME bioprinting techniques have enormous potential for creating 3D heterogeneous and physiologically-relevant tissue constructs.

## Results and discussion

### Biofabrication of liver lobule-mimetic constructs

**Figure 1:**
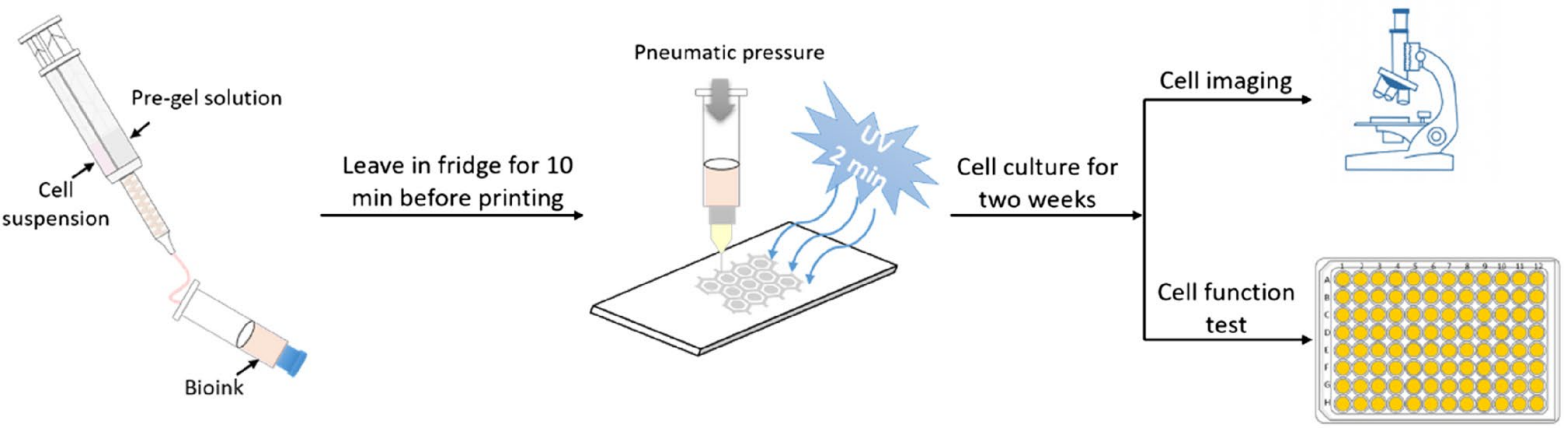
Schematic illustration of the biofabrication process.

Herein, we fabricated liver tissue-mimetic constructs according to the process illustrated in Fig. 1. To mimic an array of liver lobules to-scale, a honeycomb lattice was first printed with a bioink composed of 1% alginate, 3% CNC, and 5% GelMA (135ACG). The honeycomb lattice has a 0.48 mm wall thickness, 0.4 mm wall height, and 2.4 mm spacing. Then the inner cavities of the hexagon units were filled with 4% GelMA. Four different variations were 3D printed in this work, referred to as S1 (honeycomb: fibroblasts-laden 135ACG, middle cavities: acellular GelMA); S2 (honeycomb: acellular 135ACG, middle cavities: hepG2-laden GelMA); S3 (honeycomb: acellular 135ACG, middle cavities: fibroblasts/hepG2-laden GelMA); and S4 (honeycomb: NIH/3T3-laden 135ACG, middle cavities: hepG2-laden GelMA). The printed constructs were cultured in phenol-red free cell culture media for 2 weeks. Cell viability, proliferation, and morphology in the constructs were studied using confocal fluorescence microscopy on days 1, 4, 7, 11, and 14. Cell culturing media were collected on days 1, 4, 7, and 14 to study the cellular activities through albumin production. The four variations allowed us to compare single-cell 3D structures (S1, S2) with heterogeneous bicellular structures (S3, S4). Furthermore, S3 and S4 were designed to enable direct-contact (S3) and non-contact (S4) interactions between hepG2 and the supporting cells NIH/3T3.

### Bioink formulation and properties

**Figure 2:**
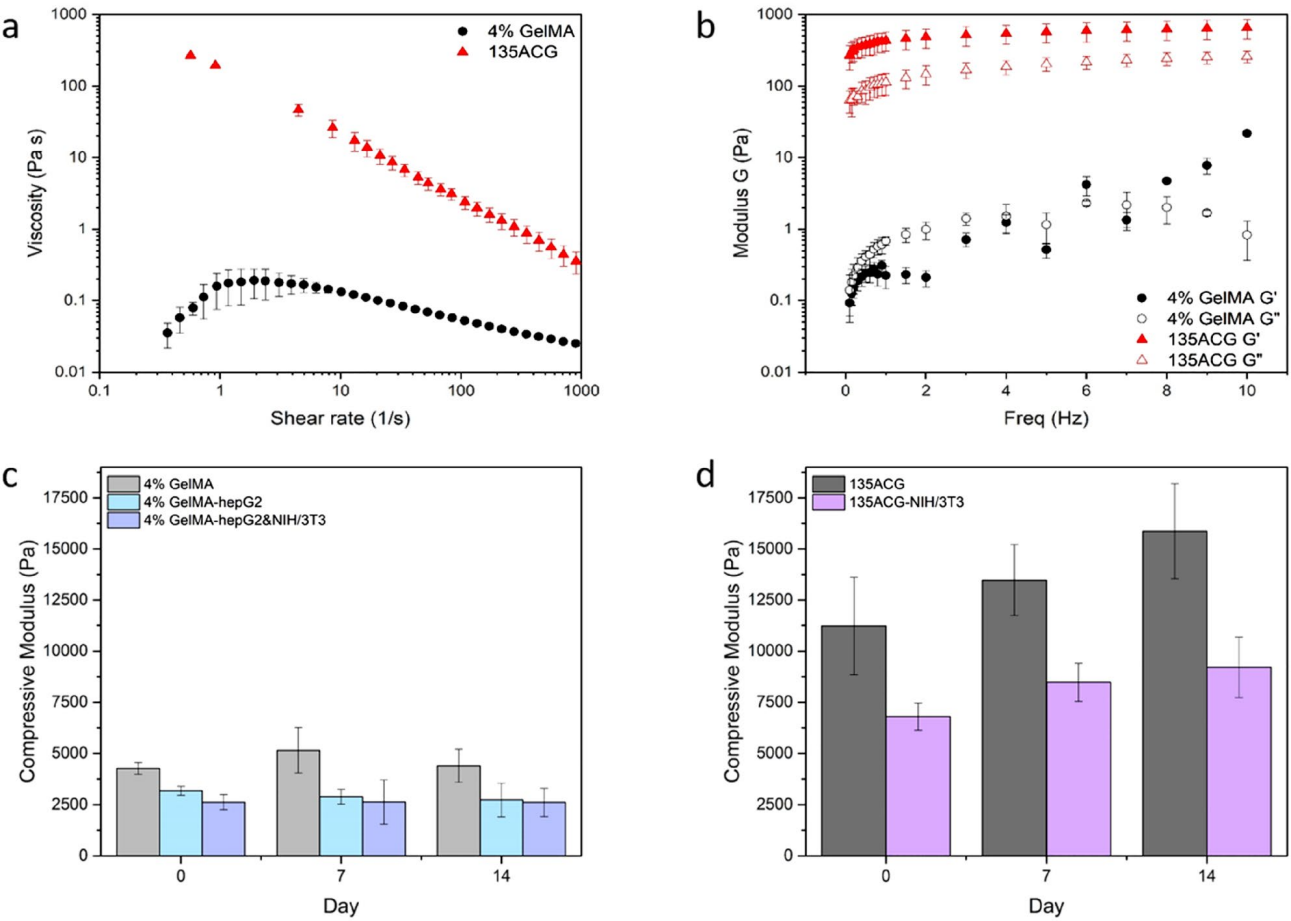
Characterization of the bioinks. (**a**) Flow curves of two different bioinks, 4% GelMA and 135ACG. (**b**) Elastic modulus (G′) and viscous modulus (G″) of the two bioinks as function of oscillatory frequency. Compression modulus changes of (**c**) 4% GelMA and (**d**) 135ACG gels with/without cells over two weeks.

To enable high-resolution bioprinting, advanced bioink formulations and cross-linking mechanisms were developed. GelMA was synthesized by converting lysine groups contained in gelatin to methacryloyl groups to allow UV crosslinking (Fig. S1) and the degree of substitution (DOS) is calculated to be around 85%, as shown in the supporting information (SI). The rheological properties of 4% GelMA and a hybrid bioink 135ACG were tested. As shown in Fig. 2a, 4% GelMA showed a slight shear-thickening behavior at low shear rates and exhibited a slight decrease in viscosity at high shear rates. But overall, its viscosity is around 0.1 Pa s and varies little over the whole shear rate range. The shear-thickening at low rates could be attributed to intermolecular interactions of GelMA^32,33^, which can cause shear thickening when the energy of the physical bond and the thermal energy are similar in magnitude^34^. Another probable cause is the experimental artifact caused by the unsteady state of low viscosity GelMA at low shearing rate^35^. The GelMA coils can disentangle and orientate in the flow direction with increasing shear rate, leading to the observed shear-thinning behavior after a critical shear rate is reached. The incorporation of CNC and alginate into the GelMA solution significantly altered the intermolecular interactions, leading to drastically different rheological properties. The hybrid bioink, 135ACG, exhibited a strong shear-thinning property over the entire shear rate range of 0.1–1000 s^−1^ with a curve slope of − 0.85. The 135ACG ink showed a viscosity close to 200 Pa s at static state and 0.2 Pa s at the shear rate of 1000 s^−1^ (i.e. 3 orders lower). At static state, alginate molecules wrap around the surface of the CNCs mainly through electrostatic interaction due to the negatively charged CNC surfaces. When a shear stress is applied, the entangled polymers re-align and lowers the bioink’s viscosity. Compared with hybrid bioinks reported previously by our group and other groups^19,36–38^, the new 135ACG bioink showed a much stronger shear thinning behavior and one order of magnitude higher viscosity at static state, indicating its superiority for ME printing. Step-shear measurements illustrated the injectability and self-healing behavior of the 135ACG bioink after being applied high shear which is essential when the bioink is extruded through a high-gauge needle (Fig. S2). Further, as shown in Fig. 2b, the viscous modulus Gʺ was higher than the elastic modulus Gʹ in 4% GelMA at low frequencies, while Gʹ overrode Gʺ at around 8 Hz, indicating that pure GelMA was more liquid-like at low frequencies and not suitable for ME bioprinting since the modulus of 4% GelMA was lower than 10 Pa. On the other hand, for 135ACG, Gʹ were higher than Gʺ over the entire frequency range, suggesting that 135ACG is more solid-like and can produce scaffolds with good structural fidelity^19^.

Besides printability, bioinks must offer scaffolds with suitable mechanical strength and degradation profile for tissue engineering. After printing, cell-laden and acellular constructs were crosslinked chemically (by UV), then cultured over two weeks. Their compressive moduli were measured on days 0, 7, 14. The compressive modulus of 4% GelMA was 4269 ± 278 Pa immediately after preparation (day 0). Upon cell addition, the overall compressive modulus reduced to 3175 ± 217 Pa (hepG2 cells only) and 2624 ± 370 (hepG2/fibroblast cell mixture), respectively (Fig. 2c). Neither acellular nor cell-laden GelMA constructs exhibited significant decrease in overall mechanical strength over the two-week period (Fig. 2c, day 7 and 14), indicating that the enzymatic degradation of GelMA and the generation of natural extracellular matrix (ECM) by embedded cells were balanced^39^. Furthermore, our data confirmed that the GelMA scaffolds can provide a stable and soft environment ideal for the growth of hepG2 cells over the entire study period. Ma et al*.* reported that the stiffness of the scaffolds influenced the hepG2 cellular growth and functions such that hepG2 cells cultured in the scaffolds with a stiffness of 0.5 kPa and 5 kPa tend to aggregate and form spheroids^29^.

The compressive modulus of acellular 135ACG scaffolds was 11,222 ± 2381 Pa on day 0 and the addition of fibroblast cells lowered the stiffness to 6800 ± 664 Pa (Fig. 2d). This is consistent with previous literature reports that, when the stiffness of the substrate at the fibroblasts attachment sites were over 5 kPa, the cells were observed to decrease overall scaffold stiffness^40^. Hence, the decreased compressive modulus induced by the incorporation of cells was likely caused by the softer cells encapsulated and the material voids^41^. Interestingly, the compressive moduli of both acellular and cellular 135ACG increased to 15,876 ± 2324 Pa and 9204 ± 1480 on day 14, respectively. The increased compressive moduli can be attributed to the strengthening of alginate network by Ca^2+^ in the cell medium.

### Printability of the hybrid bioink

**Figure 3:**
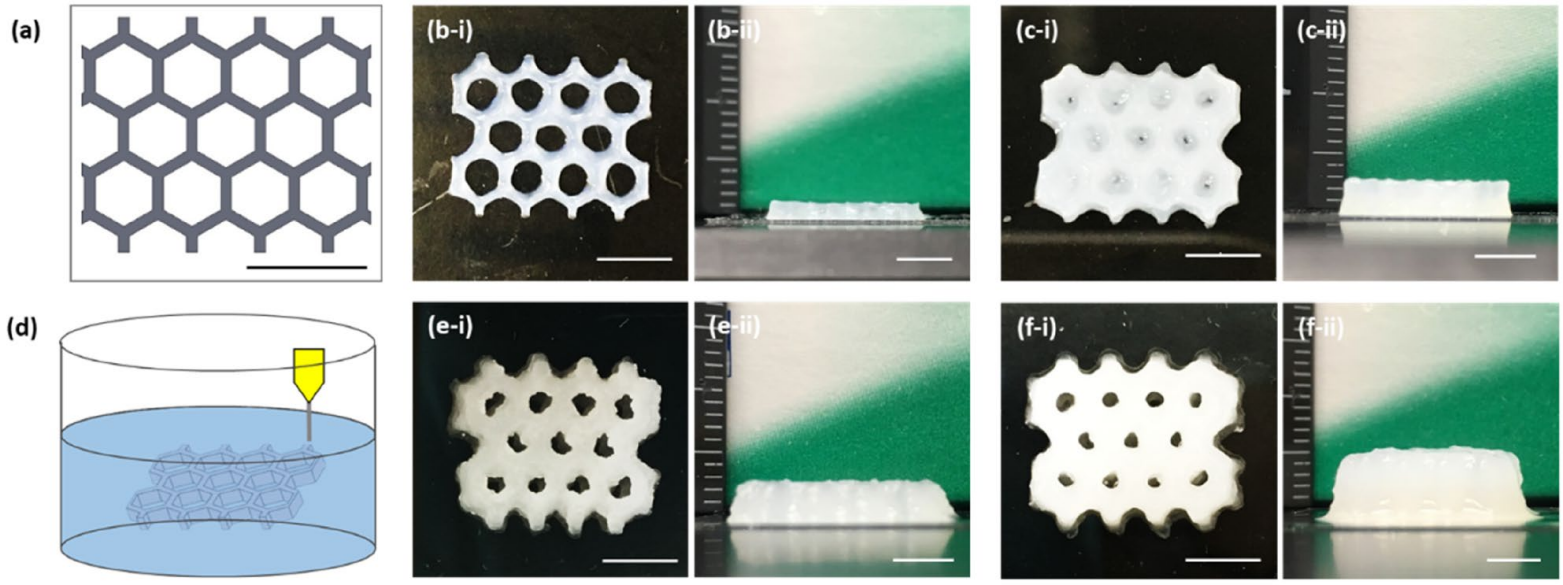
Printability of the hybrid bioink. (**a**) Schematic of the liver lobule-mimetic honeycomb structure. (**b**-I, **c**-i) Top and (**b**-ii, **c**-ii) side views of the free-form printed structures with a height of 1.8 mm and 3.4 mm, respectively. (**d**) Schematic illustration of embedded printing of the honeycomb structure. (**e**–**f**, **f**-i) Top and (**e**-ii, **f**-ii) side views of the embedded-printed structures with a height of 3.75 mm and 6.8 mm, respectively. Scale bars: 5 mm.

Honeycomb architectures, with 0.48 mm wall thickness and 2.4 mm spacing, were printed using two printing methods (Fig. 3), in order to evaluate the suitability of the hybrid ink 135ACG for printing 3D structures in terms of resolution, pattern fidelity, and geometrical aspect ratio. Free-form printing, that is to directly layer-by-layer ME printing on a cover glass, yielded structures with favorable fidelity up to a height of 1.8 mm (Fig. 3b). Upon further addition of layers, spreading and collapsing began to occur. Without in-situ crosslinking or support, ME printing using 135ACG bioink can yield honeycomb structures up to a height of 3.4 mm without completely filling up the inner cavities (Fig. 3c). This is enabled by the excellent shear-thinning and solid-like properties of the hybrid bioink, which is not achievable using pure polymeric inks at much higher concentrations. An embedded printing strategy was also developed, as illustrated in Fig. 3d. Generally, the support bath for embedded printing must be stiff enough to hold the extruded filaments in place, rapidly self-heal, and possess a low yield stress to accommodate needle movement^42,43^. Thus, a Bingham plastic material is a good candidate to act as a rigid body at low shear rates and a viscous fluid at higher shear rates^11,44^. Previously, Noor et al*.* developed a transparent support medium composed of alginate microparticles in xanthan gum solution^45^. Based on their work, we further adjusted the composition to better fit our system and rheological properties of the modified support bath material were measured. It exhibited a shear-thinning behavior, and the viscosity was approximately 50 Pa s at 0.1 s^−1^ (Fig. S3a). The elastic modulus was larger than the viscous modulus over the entire frequency, and the elastic modulus was around 100 Pa (Fig. S3b). In addition, the yielding stress of the mixture was approximately 10 Pa (Fig. S3c–d). As shown in Fig. 3e,f, honeycomb structures with a height of 3.75 mm and 6.8 mm respectively were generated using embedded-printing with better pattern fidelity than the 3.4 mm-tall structure generated by free-form printing (Fig. 3c). With embedded printing, we demonstrated that the 135ACG formulation can be used to generate tall (up to 6.8 mm), high water content (> 90%), high porosity hydrogel structures (i.e. suitable ECM), due to the enhancement of hydrogel mechanical strength by CNC incorporation. Overall, these printing results exemplified the great potential of the unique CNC-incorporated 135ACG bioink for ME printing of cell-compatible structures with high aspect ratio^46–48^.

### Cell behavior in bioprinted mono-cellular 3D constructs

**Figure 4:**
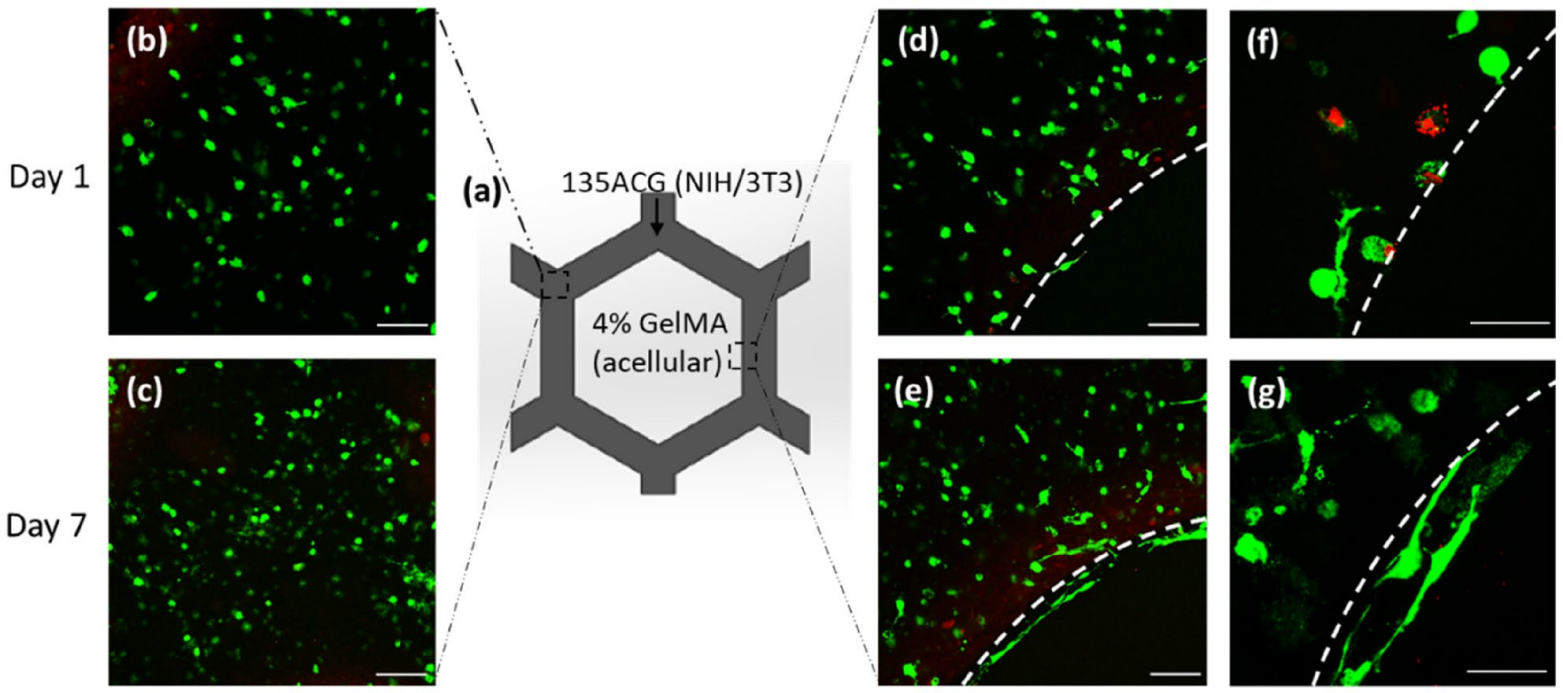
NIH/3T3 cell growth, proliferation and morphology in 135ACG. (**a**) Schematic of construct S1. Fluorescence images of NIH/3T3 on days 1 and 7 (green: live cells; red: dead cells) encapsulated in the middle of honeycomb wall (**b**,**c**), as well as at the boundary of 135ACG and GelMA (dashed line) (**d**,**e**). Zoomed-in images of NIH/3T3 at the boundary on (**f**) day 1 and (**g**) day 7. Scale bars are 100 μm in (**b**–**e**) and 50 μm in (**f**–**g**).

A mono-cellular construct with NIH/3T3 cells embedded in 135ACG (S1), as schematically illustrated in Fig. 4a, was first printed. The inner cavities of the hexagon units were filled with acellular 4% GelMA. Cells proliferated over time (Fig. 4b,c), the elongation of NIH/3T3 being observed on day 7 (Fig. 4c), confirming that the printing process did not reduce cell viability or influence cell morphology and the 135ACG gel is a suitable ECM for NIH/3T3. In comparison, fibroblasts were arrested in CNC/Alginate only (no GelMA) matrix (data not shown). Thus, GelMA provided cell adhesion sites and facilitated the proliferation of NIH/3T3 cells in the 135ACG scaffolds. In addition, cells were elongated and aligned along the boundary of the two different bioinks, which can be explained by durotaxis, a mechanism of cell migration guided by the stiffness of the ECM (Fig. 4d–g). Fibroblast cells tend to migrate from softer toward stiffer substrates because a more stable focal adhesion and higher traction forces are formed in a stiffer region^49^. Therefore, when NIH/3T3 cells migrated in the stiffer 135ACG bioink, the protrusion of the cells’ leading edge stopped at the interface of the two different bioinks due to durotaxis and continued laterally along the boundary of the two hydrogels while the trailing edge retracted, leading to the observed orientation of the cells^50,51^.

Furthermore, NIH/3T3 cells located near the bottom (cover glass) showed more elongated morphologies, while cells in the upper portion of the constructs exhibited rounder morphologies (Fig. S4). The mechanical gradient between the soft hydrogel and the stiff substrate causes an edge effect, increasing the stress on the hydrogels near the bottom of the printed scaffolds. Cells sensed the stiffness of their environment and modulated their morphology accordingly^49^.

**Figure 5:**
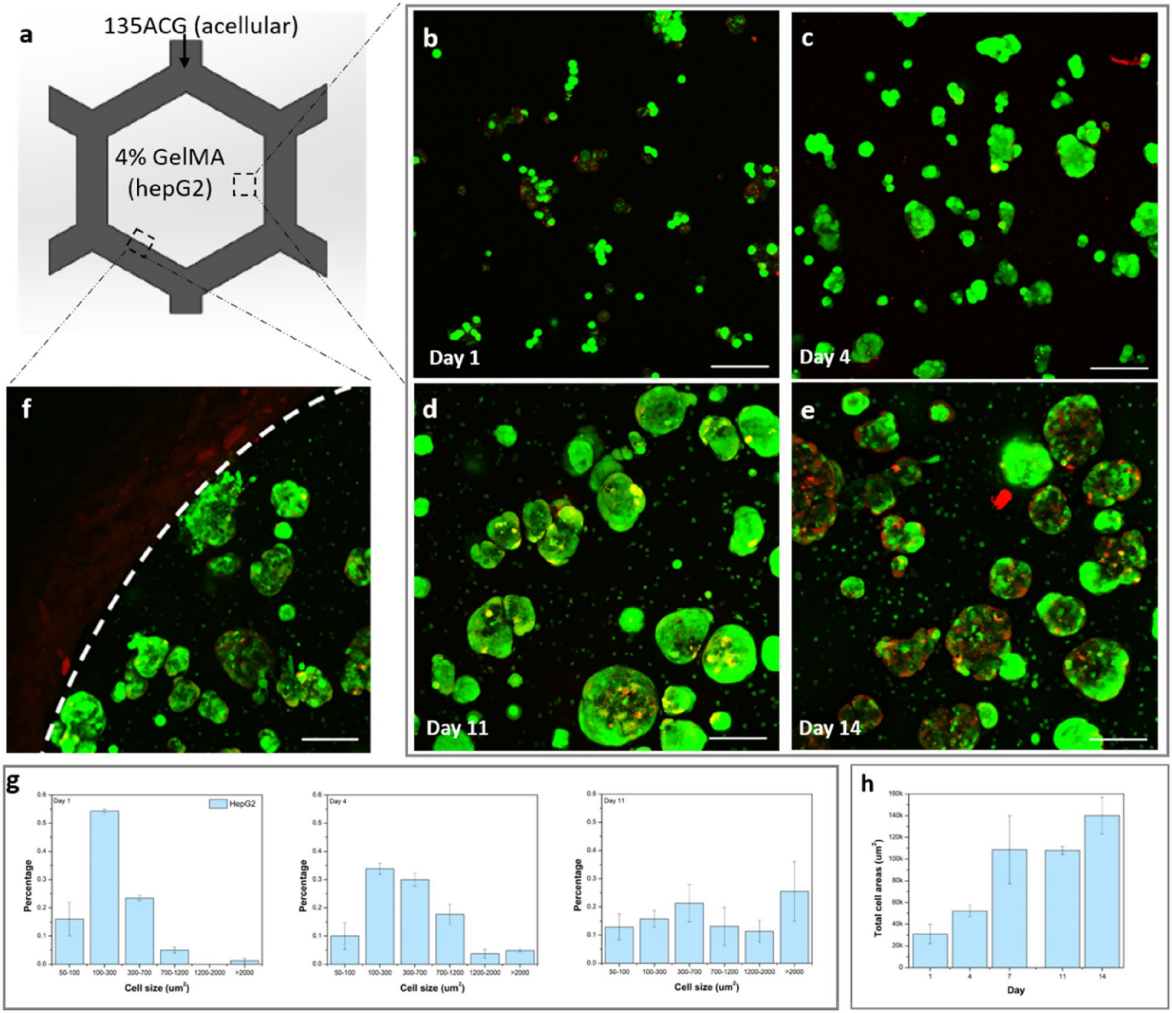
HepG2 cell growth, proliferation and morphology in GelMA. (**a**) Schematic of construct S2. (**b**–**e**) Fluorescence images of hepG2 in 4% GelMA on days 1, 4, 11, and 14 (green: live cells; red: dead cells). (**f**) HepG2 at the boundary of 135ACG and GelMA (dashed line). Scale bars are 100 μm. (**g**) HepG2 cell/ cluster area distribution on days 1, 4, and 11. (**h**) Total hepG2 cell spreading area over a 14-day period.

A second mono-cellular construct was printed with acellular 135ACG ink first and the inner cavities were filled with HepG2 cell-laden GelMA (4%) (S2), as illustrated in Fig. 5a. Cell proliferation and viabilities in 2D hepG2 and 3D (i.e. hepG2 embedded in GelMA) cultures were compared. Our results showed that hepG2 cells in 2D cultures proliferated much faster than in 3D cultures, as shown in Fig. S5a. Thus, the initial cell concentration in 2D cultures was chosen to be relatively low, at 50 K cells/well in a 6-well plate, while in 3D cultures the initial cell density was 2.5 M cells/mL. Cell viabilities in both 2D & 3D cultures were confirmed to be greater than 90% over the 14-day study period (Fig. S5b). Furthermore, hepG2 cell morphology was studied using confocal fluorescence microscopy. HepG2 cells were observed to be mostly single or in small aggregates on day 1(Fig. 5b), form spheroids four days post printing (Fig. 5c), and the spheroids increased significantly in size over time (Fig. 5c–d). High cell viability was maintained until day 11 (Fig. 5d). On days 11 and 14, dead cells were observed mainly in the centers of large spheroids (Fig. 5d,e), likely due to nutrient depletion and the accumulation of toxic products. A close examination of the boundary between the soft GelMA and the stiff 135ACG gels revealed that hepG2 propagated exclusively in the GelMA matrix (Fig. 5f). Cell/cluster area distribution and the total cell spreading areas were extracted via statistical analysis of the fluorescence images and plotted in Fig. 5g–h. On day 1, most of the cell cluster areas were in the range of 100–300 μm^2^, i.e. the size of single cells and small cell aggregates. Cell/cluster sizes were shown to steadily increase in day 4 and 11 (Fig. 5g), indicating that hepG2 cells formed larger cell clusters/spheroids over time in GelMA. However, the total cell spreading area plateaued on day 7 (Fig. 5h), which is consistent with the growth curve shown in Fig. S5a. Taken together, 4% GelMA hydrogel possessed a similar mechanical strength to the liver tissue and provided an optimal environment for the growth of hepG2 cells, which is consistent with the findings from Ma et al^29^.

### Cell behavior in bioprinted bi-cellular 3D constructs

**Figure 6:**
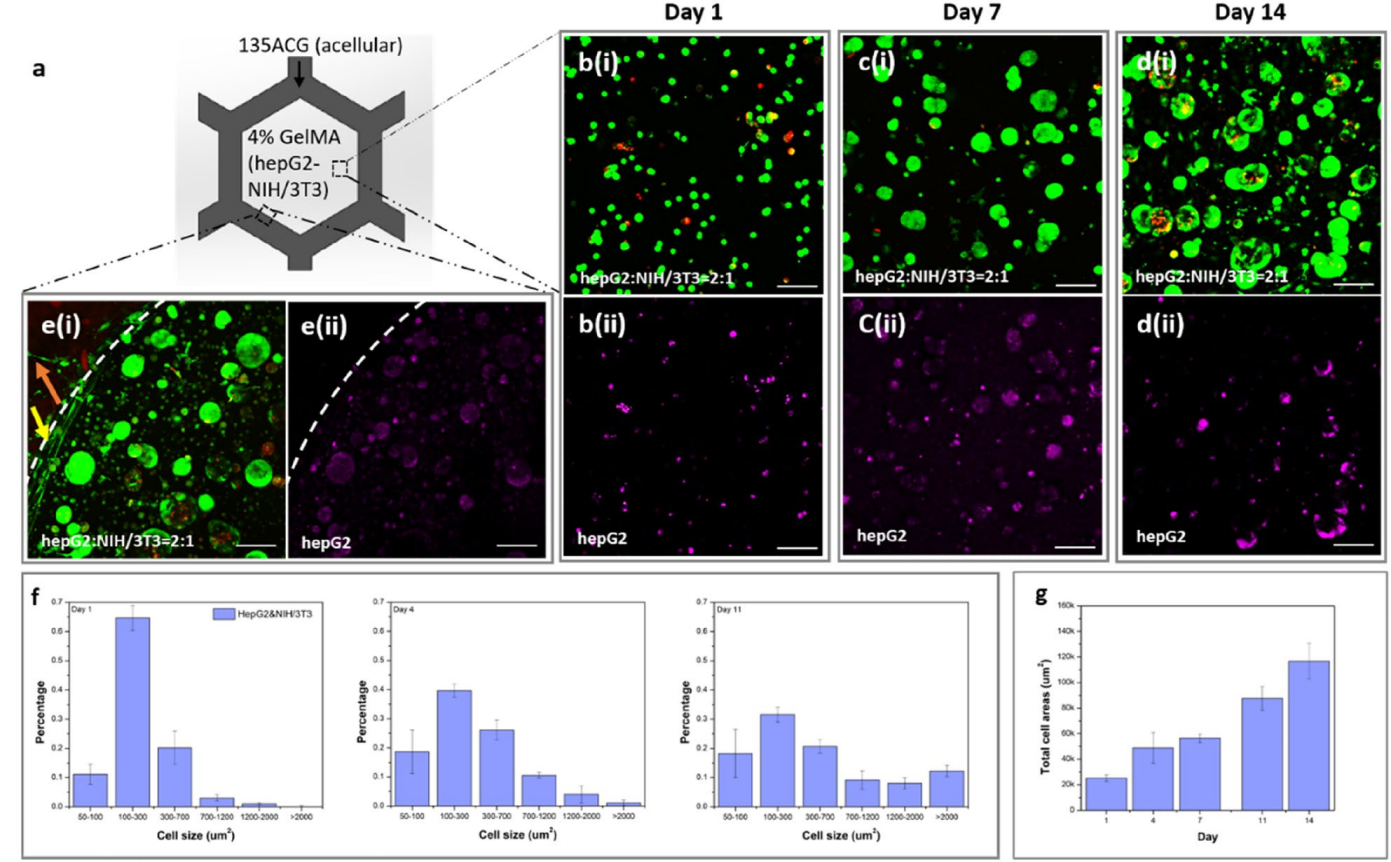
Mixed NIH/3T3 and hepG2 cell growth, proliferation, and morphology in GelMA. (**a**) Schematic of construct S3. Fluorescence images of the hepG2 and NIH/3T3 mixture (initial densities of hepG3: NIH/3T3 = 2:1) on (**b**-i) day 1, (**c**-i) day 7, and (**d**-i) day 14 (green: live cells; red: dead cells). The images of hepG2 cells stained with Qtracker 655 (magenta) on (**b**-ii) day 1, (**c**-ii) day7, and (**d**-ii) day 14. (**e**) Cells at the boundary of 135ACG and GelMA (dashed line). Scale bars are 100 μm in (**b**–**e**). (**f**) Cell/cluster area distribution in S3 on days 1, 4, and 11. (**g**) Total cell spreading area in S3 over a 14-day period.

**Figure 7:**
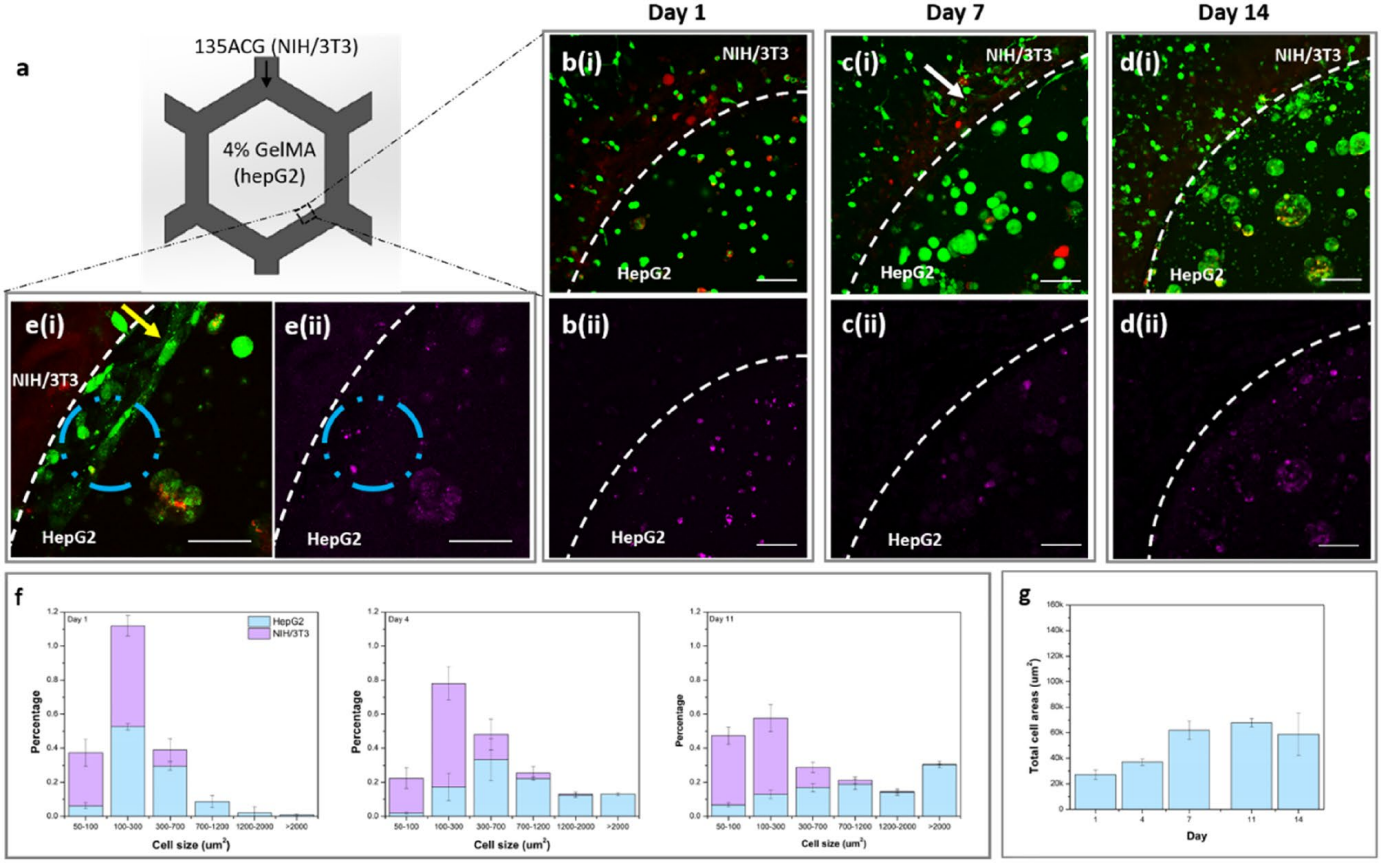
Adjacent NIH/3T3 (in 135ACG) and hepG2 (in GelMA) cell growth, proliferation and morphology. (**a**) Schematic of construct S4. Fluorescence images of both types of cells near the boundary of 135ACG and GelMA (dashed line) on (**b**-i) day 1, (**c**-i) day 7, and (**d**-i) day 14 (green: live cells; red: dead cells). Images of hepG2 cells stained with Qtracker 655 (magenta) on (**b**-ii) day 1, (**c**-ii) day 7, and (**d**-ii) day 14. (**e**) Zoomed-in images of the cells at the boundary. Scale bars are 100 μm in (**b**–**d**) and 50 μm in (**e**). (**f**) Cell/cluster area distribution of hepG2 spheroids and NIH/3T3 cells separately on days 1, 4, and 11. (**g**) Total hepG2 cell spreading area over a 14-day period.

To investigate the effect of co-culturing NIH/3T3 cells with hepG2 cells in the bioprinted constructs, systems with direct intercellular contact (S3) and non-contact (S4) co-culture systems, as described in Figs. 6a and 7a, were fabricated separately. To generate S3, acellular 135ACG honeycombs were first printed, followed by depositing a homogeneous mixture of hepG2 and NIH/3T3 fibroblasts in GelMA (4%) to fill the cavities. Both types of cells exhibited round morphology on day 1 (Fig. 6b). Similar to S2, spheroids were observed to form over time in S3 (Fig. 6b–d), but with both cell types presenting in the clusters. Elongation and spreading of individual NIH/3T3 cells was also observed in S3, as shown in Fig. 6d. The protrusion of NIH/3T3 cells into stiff 135ACG (orange arrow) and the parallel alignment of NIH/3T3 cells along the boundary of the two different matrices (135ACG and GelMA) (yellow arrow) were observed on day 14 (Fig. 6e). Cell area distribution and total spreading areas in S3 were analyzed and plotted in Fig. 6f–g. Compared to S2, there were a much lower percentage of cell/clusters with an area larger than 2000 μm^2^ and a higher percentage below 700 μm^2^ in S3, indicating that spheroids formed in the hepG2/NIH/3T3 co-culture were smaller than those in the hepG2-only culture and some NIH/3T3 cells propagated as single cells (Fig. 6f). The total cell spreading area increased persistently over the two weeks (Fig. 6g), instead of plateauing on day 7 as in S2, which is ascribed to fibroblast growth.

To fabricate S4, NIH/3T3-laden 135ACG was used to print the honeycombs and then hepG2-laden GelMA was used to fill the cavities. Interestingly, the two types of cells migrated towards each other over time (Fig. 7b–d). The spreading of NIH/3T3 cells in 135ACG (white arrow) was observed on day 7 (Fig. 7c), aligning parallel to the boundary of the two bioinks on day 14 (Fig. 7e). Moreover, direct intercellular contact of the two types of cells at the boundary occurred as shown in Fig. 7e labeled with a blue circle. The cell size distribution of NIH/3T3 and hepG2 cells at the boundary region were analyzed separately and plotted as stacked columns in Fig. 7f–g. The trend of the hepG2 cell/cluster area distribution in S4 (Fig. 7f) was the same as that in S2. In contrast to hepG2, the size of fibroblasts was smaller, mainly in the range of 50–700 μm^2^, and did not significantly change over time. It can be explained by the fact that NIH/3T3 cells grew in the form of single cells and did not aggregate over time. Moreover, the total hepG2 cell spreading area in S4 plateaued on day 7 (Fig. 7g).

### Albumin secretion

**Figure 8:**
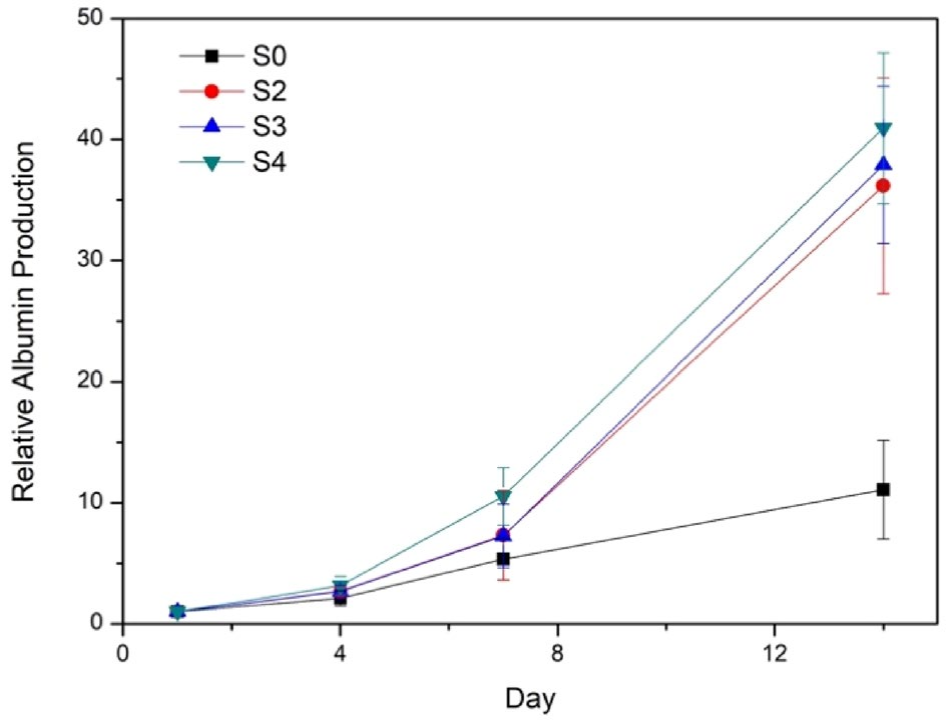
Relative albumin production by hepG2 cultured in 4 different systems: S0 (2D), S2, S3, and S4 on days 1, 4, 7, and 14.

One of the most important functions of hepatocytes is the synthesis of serum protein, specifically serum albumin. Thus, to assess hepG2 cell function in 3D mono-cellular (S2) and bicellular co-culture systems (S3, S4), levels of albumin production were measured over two weeks and compared to that in a 2D system (S0). In each culture, the amount of albumin produced was measured every 24 h and plotted in relative to the level on day 1 (Fig. 8). Albumin secretion hiked up dramatically in all 3D cultures starting on day 7 and reached about 35–40 times on day 14, even though hepG2 proliferated much faster in a 2D culture, and cell numbers in 3D cultures plateaued on day 7 (Fig. S5a). This result indicates that, compared to culturing in 2D, encapsulation of hepG2 in the 3D GelMA ECM promoted per-cell albumin secretion significantly. From day 7 to 14, higher amount of albumin was produced from the co-culture system which could be explained by the presence of NIH/3T3 fibroblasts. Among the 3D systems, albumin secretion was slightly higher in the hepG2/NIH/3T3 co-cultures (S3, S4) than that in the hepG2-only culture (S2), consistent with literature data. Previous studies have shown that hepatocytes co-cultured with non-parenchymal cells in a 3D environment exhibited enhanced liver functions, such as albumin secretion and urea production, when compared to the traditional 2D and mono-cellular 3D culturing techniques^52–54^. Jeong et al*.* have found that rat albumin secreted by fibroblasts in cell media was negligible^55^, meaning that albumin was only produced by hepG2 cells. Interestingly, there was no significant difference in the level of albumin production in the co-cultures with direct (S3) and in-direct intercellular contacts (S4), suggesting that the physical contact of the dual cell lines might not be necessary to influence the hepatocyte function, rather the fibroblast-secreted factors matter^55^.

### Conclusion

We have developed two types of bioinks and methods suitable for the ME printing of heterogeneous 3D constructs. A CNC-incorporated alginate/GelMA hybrid bioink (135ACG) was formulated for precision printing of high aspect ratio cell-laden structures. Due to the excellent shear-thinning and solid-like properties of 135ACG, honeycombs (with a 0.48 mm wall thickness) up to 3.4 mm and 6.8 mm were demonstrated with free-form printing and embedded printing respectively. After UV crosslinking, scaffolds printed with 135ACG were also confirmed to offer a stiff ECM, with a compressive modulus on the order of 10 kPa, to accommodate the adhesion, spreading, and proliferation of fibroblasts, an example stromal cell line. A softer GelMA hydrogel that possessed stiffness (compressive modulus ~ 2.5 kPa) comparable to the native liver tissue was used to encapsulate hepatocytes (hepG2). Four different 3D liver lobule-mimetic structures with precise placement of hepG2 and NIH/3T3 cells were bioprinted. The four structures allowed us to observe effects of mechanical cues and intercellular interactions on cell behaviors, including hepatic spheroid formation, cell alignment and migration at the boundaries of stiff/soft ECMs, as well as albumin secretion, the most important function of hepatocytes. Interestingly, fibroblasts thrived in stiff 135ACG matrix and aligned at the 135ACG/GelMA boundary due to durotaxis, while hepG2 formed spheroids exclusively in the soft GelMA matrix. Hepatic spheroids formed by a homogeneous mixture of hepG2/NIH/3T3 were found to be smaller than those formed by hepG2 only. Improved albumin production was observed when hepG2 cells were co-cultured with NIH/3T3 both with and without direct intercellular contact, indicating that improved hepatic function can be attributed to soluble chemical factors. Overall, our results demonstrated the great potential of the bioink materials and ME-based printing methods developed in this study, which offered new strategies to address the central challenge in tissue engineering to create complex constructs with multiple cell types and varying ECMs to recapitulate biological functions.

## Methods

### Materials

Sodium carbonate, sodium bicarbonate, gelatin (type A, 300 g Bloom, from porcine skin), deuterium oxide (D_2_O), methacrylic anhydride (MAA), calcium carbonate, D-( +)-gluconic acid δ-lacton, and lithium phenyl-2, 4, 6,-trimethylbenzoyphosphinate (LAP) were purchased from Sigma Aldrich. Xanthan gum was purchased from CP Kelco. Pharmaceutical grade sodium alginate (PROTANAL LF 10/60 FT) with 60–70% guluronate (G) residues was acquired from FMC (Philadelphia, PA). Dulbecco's Modified Eagle's Medium (DMEM), fetal bovine serum (FBS), trypan blue stain (0.4%), and OmniPur 10X phosphate buffered saline (PBS) concentrate were purchased from VWR. Penicillin/streptomycin, TrypLE Express, LIVE/DEAD viability/cytotoxicity kit, trypsin/EDTA solution, and Qtracker 655 cell labeling kit were purchased from Thermo Fisher. Human albumin ELISA kit was purchased from Abcam. Cellulose nanocrystals were donated by Professor Michael K.C. Tam from the Department of Chemical Engineering at the University of Waterloo.

### GelMA synthesis

GelMA was synthesized with the method developed by Shirahama et al^56^*.* In brief, gelatin solution (10%) was obtained by dissolving gelatin power in carbonate-bicarbonate (CB) buffer and adjusted to pH 9.0 in round bottom flask at 50 °C. MAA (0.1 mL MAA/1 g gelatin) was added dropwise. After three hours of reaction, the reaction mixture was centrifuged at 3250 g for 10 min and adjusted to pH 7.4. The solution was diluted with double the volume of DI water and dialyzed with a 12 kDa molecular-weight-cutoff (MWCO) membrane against DI water at 50 °C for one week, followed by flash frozen in liquid N_2_ and lyophilized. The GelMA product was stored in the dark at − 20 °C. To determine the degree of substitution (DOS), the lyophilized GelMA was dissolved in D_2_O at 50 mg/mL and analyzed via proton nuclear magnetic resonance spectroscopy (^1^H-NMR).

### Bioink preparation

CNC powder was suspended in Milli-Q water at a concentration of 6 wt% and sonicated for 10 min at 37 kHz. Alginate (2 wt%) was then added to the mixture and vortexed for 5 min followed by centrifugation at 2000 g for 3 min. The resulting mixture was then vortexed and centrifuged again, followed by 10 min of sonication at 37 kHz. GelMA (10%) was dissolved in 2X PBS containing 0.2 wt% LAP. For the rheological measurements, the two different solutions were mixed at the same volume to reach a final concentration solution of 1 wt% alginate, 3 wt% CNC, and 5 wt% GelMA containing 0.1 wt% LAP, referred to as 135ACG, vortexed for 5 min, and centrifuged for 3 min twice. GelMA (4 wt%) was prepared by dissolving GelMA in 1 $\times$ PBS containing 0.1 wt% LAP.

To make cell-laden bioink, the initial concentrations of all polymer solutions were prepared at 1.25 times the concentrations shown above. The GelMA solution was sterilized through a 0.22 μm filter. The alginate and CNC mixture was autoclaved at 121 °C for 20 min. Cell-laden GelMA (4%) solutions were obtained by mixing GelMA with cells via pipetting. Cell-laden 135ACG bioink was prepared by mixing the hydrogel and concentrated cell suspensions with a double-syringe mixer at a volume ratio of 4:1. The produced 135ACG bioink had a NIH/3T3 fibroblast cell density of 5 million per milliliter. The mixing procedure had no significant influence on the cell viability (data not shown).

### Preparation of supporting medium for embedded 3D printing

The support bath medium for printing was prepared according to the procedure developed by Noor et al^45^. Briefly, sodium alginate (0.32 wt%), Xanthan gum (0.25 wt%), and calcium carbonate (9.56 × 10^−3^ M) were dissolved in DI water and homogenized. Concentrated D-( +)-gluconic acid δ-lactone solution was added to the mixture to reach a final concentration of 19.15 mM and stirred constantly until the viscosity of the mixture increased and no precipitation of the calcium carbonate was observed. The mixture was then left at room temperature for 24 h. DI water was added to the mixture (4:1 volume ratio), homogenized, centrifuged at 11,000 g for 20 min. The precipitated pellet was collected. Xanthan gum (1 wt%) was freshly prepared and added to the pellet at a volume ratio of 1:1. A homogeneous support bath was obtained through vigorous vortexing.

### 3D printer and printing procedure

A FlashForge Creator Pro (FlashForge, China) mounted with a custom-made syringe holder was used. The syringe was connected to an Ultimus V high precision dispenser (Nordson EFD, USA). 3D models were designed using SolidWorks. Bioinks were loaded into 10 mL pneumatic syringes and refrigerated for 10 min before printing to further increase viscosity, taking advantage of the temperature responsive GelMA. Structures were printed on cover glasses directly or in the support bath with a 32-gauge nozzle at a printing speed of 20 mm/s and under an air pressure of 20 psi. After printing, the structures were then crosslinked under UV light (365 nm) for 2 min. The embedded 3D-printed constructs were washed with DI water to remove excess support bath materials.

### Measurement of bioink rheological properties

The rheological measurements were conducted using a Bohlin-CS Rheometer with a cone-plate geometry (CP 4-40). ACG hybrid bioinks were refrigerated for 10 min before measurements at room temperature. The steady-state shear viscosities were measured with a shear rate in the range of 0.1–1000 s^−1^. A strain-sweep test with a strain from 0.05 to 10% and a frequency at 1 Hz was performed to determine the linear viscoelastic regions. The optimal strain was chosen to be 0.1% for the oscillation frequency test from 0.1 to 10 Hz. Step-shear measurements were performed at 0.012 s^−1^ and 100 s^−1^.

### Measurement of scaffold stiffness

Cylindrical PDMS molds with a diameter of 6 mm and a height of approximately 2 mm were made. The acellular and cell-laden bioinks were added into the molds and crosslinked prior to uniaxial compression tests. The initial 10% strain region of a strain–stress curve was chosen to determine the compressive modulus of each sample. To gauge degradation over time, samples were cultured under standard cell culture conditions (see below) for 2 weeks. Compressive modulus of each sample was measured immediately after fabrication, on day 7, and on day 14.

### Cell culture and characterization

Fibroblasts (NIH/3T3) and liver hepatocellular carcinoma (hepG2) cells were cultured in DMEM supplemented with 10% FBS and 1% penicillin/streptomycin and maintained in a 5% CO_2_ incubator at 37 °C. For fluorescence imaging, with a Zeiss LSM 700 confocal microscope (Carl Zeiss AG, Germany), cells were stained using a LIVE/DEAD viability/cytotoxicity kit or Q-tracker following manufacturer’s instructions. The images were analyzed using ImageJ. Cell area distribution and total cell spreading areas were extracted from 6 replicates (n = 6) and are presented as mean ± standard deviation. Differences between samples were determined from the independent *t*-test and were considered statistically significant when p < 0.05. To evaluate HepG2 cell proliferation and viability inside a hydrogel matrix, cells were extracted by dissolving the matrix using TrypLE Express and then counted using a hemocytometer.

### Measurement of albumin production

Cell-laden scaffolds were cultured in 6-well plates, with 1.2 mL phenol-red free DMEM per well. Cell medium was collected from each well on days 1, 4, 7, and 14 and stored in liquid nitrogen. The cell medium was changed every other day and 24 h before being collected for albumin production test. A Human albumin ELISA kit was used to measure albumin concentrations according to manufacturer’s instructions.

## Supplementary information

Supplementary Information.
