# Supplementary material for: 3D bioprinting of bicellular liver lobule-mimetic structures via microextrusion of cellulose nanocrystal-incorporated shear-thinning bioink
Source: Sci Rep. 2020 Nov 26;10:20648. doi: 10.1038/s41598-020-77146-3 (PMC7691334; doi:10.1038/s41598-020-77146-3)
Supplement: Supplementary file 1 — Supplementary Information. [file 41598_2020_77146_MOESM1_ESM.pdf]

3D bioprinting of bicellular liver lobule-mimetic structures via microextrusion of cellulose nanocrystal-incorporated shear-thinning bioink

Yun Wu[1], *Andrew Wenger*[1], *Hossein Golzar*[1], *Xiaowu (Shirley) Tang*[1,*]

[1]Department of Chemistry & Waterloo Institute for Nanotechnology, University of Waterloo, Canada, N2L 3G1.

1. GelMA characterization

The synthesis of gelatin methacryloyl (GelMA) was confirmed through ^{1}H−NMR spectroscopy. As seen in Figure S1, there are four distinctive peaks at δ2.8 ppm, 5.5 ppm, 1.9 ppm, and 7.2 ppm, which can be attributed to methylene protons of unreacted lysine groups (i), acrylic protons of methacrylamide grafts on lysine groups (ii) and hydroxyl lysine groups (iii), methyl protons of methacrylamide grafts (iv), and aromatic groups (v), respectively. The decreased signal in peak (i) was observed in the spectrum of GelMA, indicating the successful conversion of lysine to methacryloyl functional groups. The degree of substitution (DOS) was calculated from the ratio of the integrals of the unreacted lysine groups in GelMA and the lysine groups in gelatin after normalization to the aromatic peaks. The DOS of the GelMA used in this study is 85.33%, which is in agreement with the reported data by Shirahama *et al.*[51]

$$\mathrm{DOS} = 1 - \frac{I_{GelMA}}{I_{Gelatin}} = 1 - \frac{0.1092}{0.7443} = 85.33\%$$

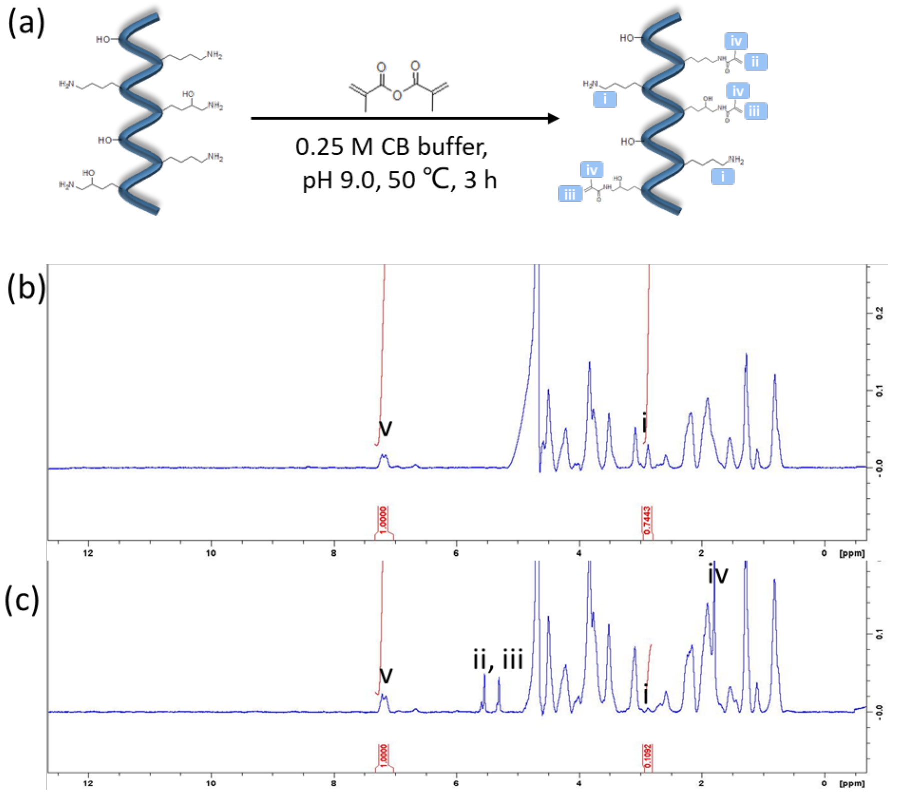

Figure S1. (a) Schematic illustration of GelMA synthesis process. ^{1}H NMR spectra of (b) gelatin and (c) GelMA.

2. Injectability and recovery behavior of 135ACG

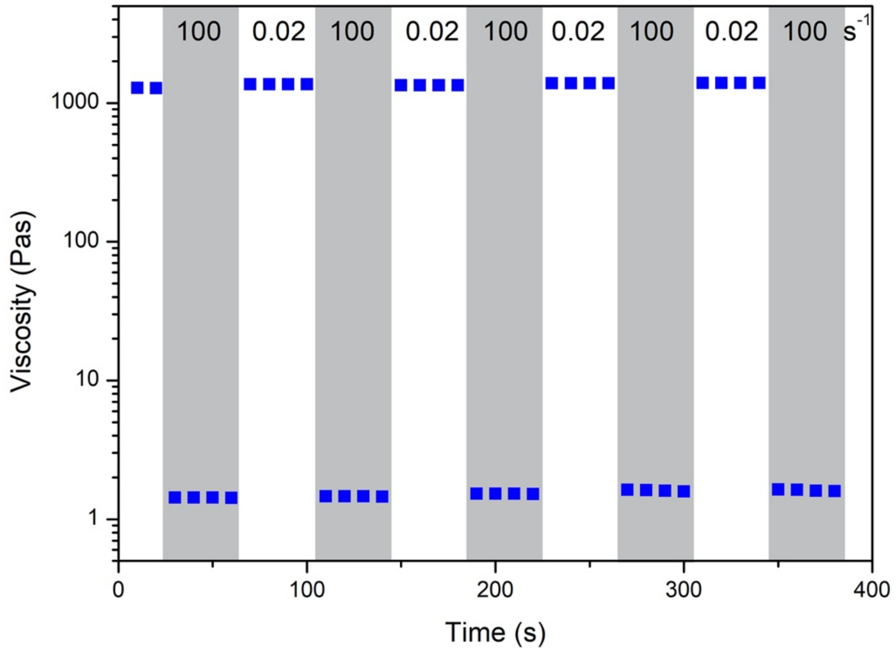

Figure S2. Step-shear measurements of 135ACG at high and low shear rate to illustrate the injectability and recovery behavior.

3. Rheological properties of the support bath material for embedded printing

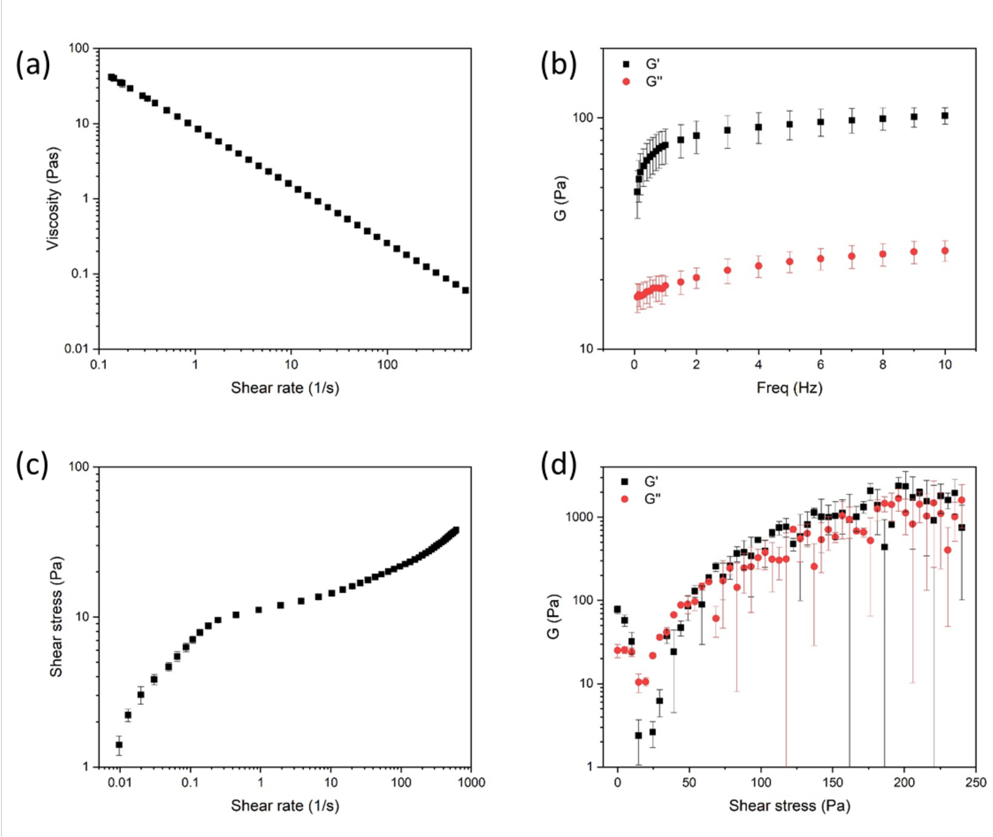

Figure S3. (a) Flow curves, (b) elastic modulus (G') and viscous modulus (G'') as a function of frequency, (c) yielding stress, and (d) elastic modulus (G') and viscous modulus (G'') as a function of shear stress of the support bath material.

4. NIH/3T3 cell morphology

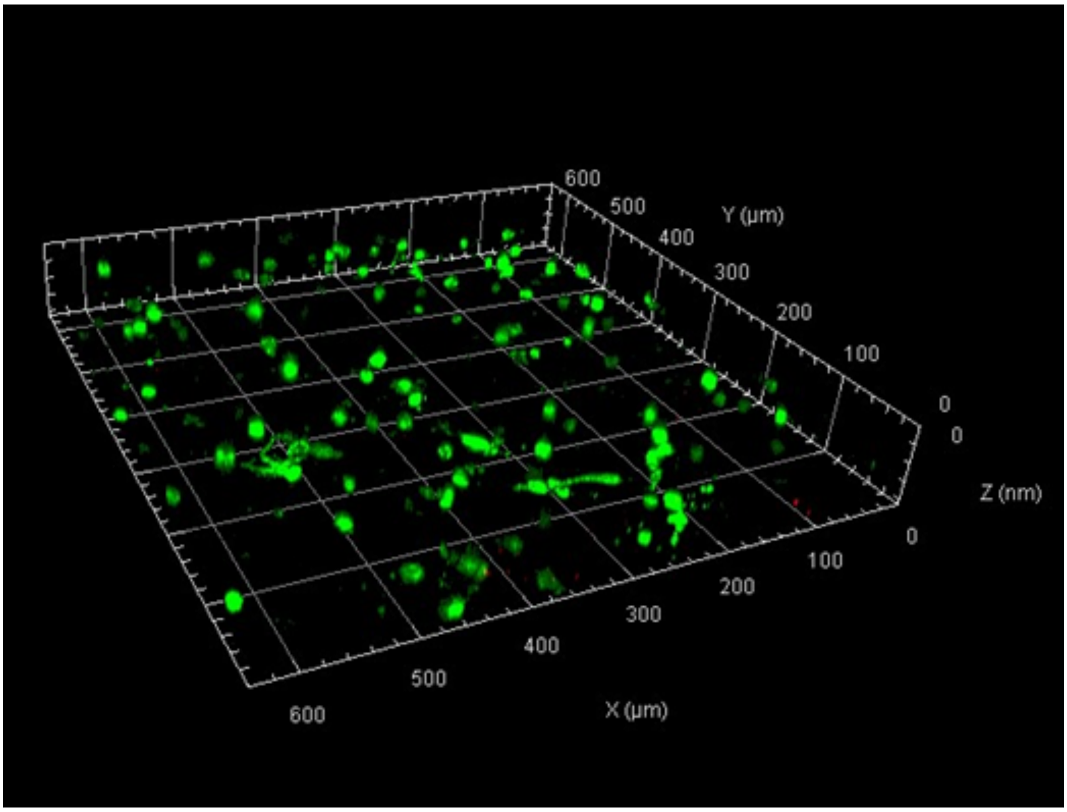

Figure S4. 3D rendered confocal fluorescence image of NIH/3T3 cells embedded in 135ACG.

5. HepG2 cell viability and proliferation

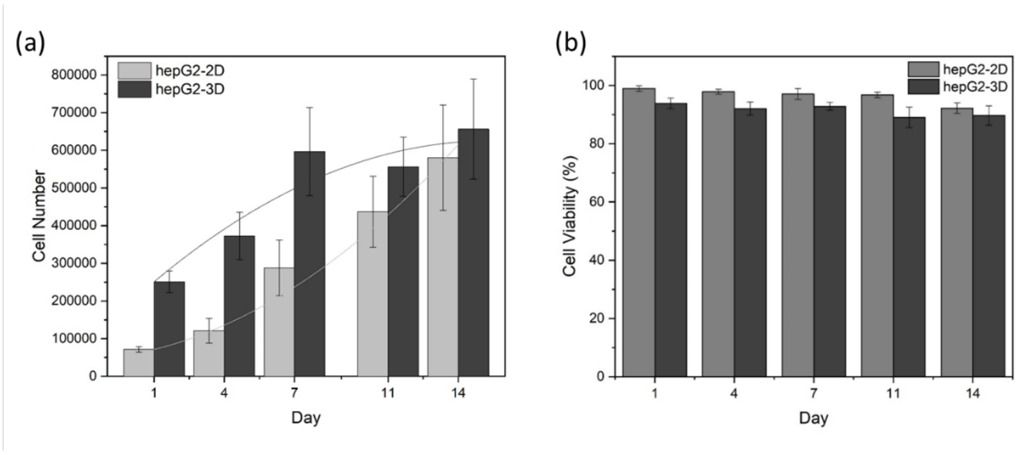

Figure S5. (a) cell number and (b) cell viability in 2D and 3D hepG2 cultures over two weeks. For 3D culture, hepG2 cells were embedded in a GelMA (4%) matrix.
